# Help‐seeking and access to care for stroke and heart attack during the COVID‐19 pandemic: A qualitative study

**DOI:** 10.1111/1467-9566.13848

**Published:** 2024-09-20

**Authors:** Christina Weis, Georgia Spiliopoulos, Agnieszka Ignatowicz, Simon Conroy, Russell Mannion, Daniel Lasserson, Carolyn Tarrant

**Affiliations:** ^1^ School of Allied Health Sciences Centre for Reproduction Research De Montfort University Leicester UK; ^2^ Department of Population Health Sciences George Davies Centre University of Leicester Leicester UK; ^3^ National Institute for Health and Care Research (NIHR) Greater Manchester Patient Safety Research Collaboration (GM PSRC) School of Health Sciences, Faculty of Biology Medicine and Health University of Manchester Manchester UK; ^4^ Murray Learning Centre College of Medical and Dental Sciences Institute of Applied Health Research University of Birmingham Birmingham UK; ^5^ MRC Unit for Lifelong Health and Ageing at UCL University College London London UK; ^6^ School of Social Policy HSMC Park House University of Birmingham Birmingham UK; ^7^ Warwick Medical School University of Warwick Coventry UK; ^8^ Department of Geriatric Medicine Oxford University Hospitals NHS Foundation Trust John Radcliffe Hospital Oxford UK

**Keywords:** candidacy, COVID‐19, England, healthcare access, heart attack, Midlands, moral work, resilience, risk, stroke

## Abstract

In this article we explore how people who experienced a stroke, transient ischaemic attack, or heart attack sought health care during the COVID‐19 lockdown periods. Semi‐structured interviews were conducted with 27 patients admitted to hospital between March 2020 and May 2021, and one carer who was recruited from cardiac and stroke rehabilitation services in two large acute NHS trusts in England. Drawing on concepts of candidacy, illness and moral work, we discuss how people’s sense‐making about their symptoms fundamentally shaped both their decisions about seeking help and the impact of COVID‐19 on help seeking. Risk perception and interactional ritual chain theory allow further exploration of constructing symbols of national identity in times of crises, managing risk and levels of acceptable risk and critique of ambiguous national messaging over accessing health‐care services for people with emergency health‐care needs. Our findings have wider implications for supporting access into health care for those with life‐threatening conditions under highly publicised strain on the health system, including winter pressure and staff strikes, as well as policymaking and public messaging.

## INTRODUCTION

The COVID‐19 pandemic was a significant shock to the health‐care system, requiring rapid and substantial changes to the organisation and delivery of emergency care to enable sufficient capacity to treat patients with COVID‐19. While the priority for the NHS was to treat and avoid mortality from COVID‐19, there was a corresponding need to ensure the provision of essential emergency care for patients with other life‐threatening non‐COVID conditions, including stroke and heart attack.

In the UK around 100,000 people have a stroke each year (Stroke Association, 2023b), and a similar number are hospitalised following a heart attack. A substantial number of excess deaths from cardiovascular disease were identified as occurring during the peak of the COVID‐19 pandemic (BHF, 2023; Wu et al., 2021). Quality of in‐hospital care for patients with acute cardiovascular disease remained consistently high during the pandemic (Douiri et al., 2021; Mohamed et al., 2021), suggesting that the adverse outcomes were unlikely to have arisen from failures in health‐care delivery, in terms of re‐organising and restructuring resources and practices to adapt to demand (Anderson et al., 2020). One contributing factor to these adverse outcomes may have been shifts in the way that the public engaged with emergency care during the pandemic.

During the earlier stages of the pandemic, reduced attendance at emergency departments and associated risks of contagion, were delaying people seeking help or facing challenges navigating access to emergency care. A survey undertaken by the UK Stroke Association (2023a) indicated that around 30% of those who had a stroke during the COVID‐19 pandemic delayed seeking emergency medical attention. Emergency ambulance calls were significantly reduced in the early stages of the pandemic (Charlton et al., 2021), and admissions for heart attack and stroke declined significantly, although some research studies have countered this (Holmes et al., 2020). There was also evidence of a rise in deaths from acute cardiovascular causes outside of hospital (Charlton et al., 2021), indicating that some people were not seeking emergency help promptly for their symptoms.

A number of studies in the US and Europe identified factors which led to delays in seeking health care during the COVID‐19 pandemic among heart attack survivors, including the belief that the health‐care system was already overburdened, fears of infection and confusion over symptoms as related to the virus and not myocardial infarction (MI) (Granström et al., 2023; Hammad et al., 2021; Lidin et al., 2021). However, when threat from MI was perceived as critical, people had less concern about COVID‐19 and were less likely to delay (Granström et al., 2023). Additional factors among heart attack survivors for delaying emergency care seeking in the UK were relying on kin and community networks and the impact of media and public health campaigns (Ferry et al., 2021), complications in accessing health care and lack of awareness of symptoms (Burton et al., 2022). Research with stroke/transient ischaemic attack (TIA) patients identified similar issues (Aref et al., 2021; Hoyer et al., 2020). The COVID‐19 pandemic added to the existing barriers experienced in accessing health care for stroke and heart attack by minority and vulnerable groups (Czeisler et al., 2020), exacerbating structural inequalities (Schuster, 2021).

The socio‐political context must be considered in relation to urgent care seeking during the pandemic. In March 2020, in response to the rising numbers of COVID‐19 cases and in order to reduce transmissions, the UK government urged the public to ‘Stay Home, Protect the NHS, Save Lives’ and ordered a first national lockdown (Tan et al., 2021). This lockdown came into force on 26 March, lasted until July 2020, and was followed by two national lockdowns, in late 2020 and early 2021, along with additional local lockdown measures in parts of England, such as the Midlands (Haddon et al., 2021), where the study participants resided and accessed health‐care services. These measures lasted for many months, causing prolonged anxiety and fears over the transmission of the disease for some and confusion over adhering to lockdown rules for others.

As part of a wider study on resilience in emergency care, we aimed to understand more about patient and carer experiences of seeking health care for symptoms of stroke and heart attack during the COVID‐19 pandemic.

### Theoretical framing

Research into health care‐related help‐seeking has a long history, identifying individual and social factors that influence help‐seeking decisions and actions. These include: symptom appraisal and perceived symptom risk, health beliefs, demographic characteristics, social support and social networks, and knowledge of and perceived appropriateness of different types of health services (Lecouturier et al., 2010; Mackenzie et al., 2013; Pfeffer, 2004).

The construct of candidacy (Dixon‐Woods et al., 2006; Tookey et al., 2018) provides a useful conceptual framing for studying the process of accessing health care. Candidacy ‘describes the ways in which eligibility for medical attention and intervention is jointly negotiated between individuals and health services’ and can be understood as ‘a dynamic and contingent process constantly being defined and redefined through interactions between individuals and professionals’ (Dixon‐Woods et al., 2006, p. 7). Candidacy is seen as ‘a process punctuated by various demands and tasks, which begins with identification of the need for professional advice or health care and culminates in adjudication from health professionals’ (Macdonald et al., 2016, p. 102). This framework has been further elaborated to illustrate a series of stages for successful health‐care provision: patients’ own sense of candidacy for medical attention; navigating health‐care services; accessing these services and patients’ experience of how ‘permeable’ these are; adjudication by health‐care professionals; resistance to accepting health‐care provision; and societal and macro‐level factors which will impact candidacy, such as availability of resources and relationship building with health‐care providers (Tookey et al., 2018). Negotiations about accessing health‐care are highly contextual and relate not only to patients’ own sense‐making of symptoms and the availability and permeability of services, but also health‐care professionals’ adjudications and subsequently patients’ fears of being rejected by services or stigmatised (Liberati et al., 2022).

A more recent model of help‐seeking investigates the complexities between sense‐making and help‐seeking behaviours specifically in seeking urgent care (Turnbull et al., 2019). This model presents a typology of ‘urgent care work’. ‘Illness work’ entails sense‐making of symptoms, and the level and type of support needed. ‘Moral work’ involves making choices about accessing appropriate services, backed by legitimate reasons for using these services, acting responsibly and finding a ‘balance between moral positioning against health risk’ (p. 4). Finally, ‘navigation work’ comprises actions such as choosing appropriate and accommodating services, and deciding how to use these, often with the assistance of social networks. This model places much emphasis on socio‐temporal contexts and therefore allows for the examination of the pandemic as an additional complicating factor in the assessment of risk.

Risk cannot be divorced from political and cultural narratives (Wilkinson, 2009). Literature on risk management and related policymaking during the COVID‐19 pandemic has focused on issues such as the importance of symbolism to create solidarity and provide legitimacy to government decision‐making, and therefore conformity (Ma et al., 2024). We discuss societal notions of risk and related national messaging, conformity being contingent on morality and shame, by using the works of Douglas and Wildavsky (1983) and Collins (2004), as expanded by King (2019). We add to this literature on individualising risk by focusing on how national messaging can prove to be detrimental to persons with urgent health‐care needs in times of crisis, when using symbolism (here the protection of the National Health Service) to ensure conformity. For Douglas and Wildavsky (1983), ‘[r]isk should be seen as a joint production of knowledge about the future and consent about the most desired prospects’ (p.5, emphasis in the original). Understanding risk and acceptable levels of risk are products of social processes. The authors emphasise the importance of politics in managing risk as a society, as social organisations formulate perceptions and create ‘cultural bias’ (p.8) surrounding risk selection and perception. The COVID‐19 pandemic acts as an illustrative example of constructing a political message based on shared values and beliefs which would unify the country, such as ‘Stay home, protect the NHS, save lives’ (Gov.uk, 2021), with the NHS considered a national symbol. However, the brunt of decision‐making was placed on individuals who had to decide on the acceptable level of risk for themselves, their kin and communities. Furthermore, in making rational decisions in uncertain times, Douglas and Wildavsky (1983) discuss ‘prospect theory’, whereby:

decisions are not focused upon final outcomes but upon incremental stages in complex processes. Stage by stage, what has gone before is treated as a boundary behind which one need not look for making the next decision; what lies two steps ahead is similarly treated as irrelevant.

(p. 78)

This approach to decision‐making in times of crises illustrates that decisions may not appear as expected under duress because of the process of breaking down a problem in its components and not considering the problem in its entirety. Additionally, individuals act according to the values and expectations prescribed in social institutions, but in their day‐to‐day living they incorporate ‘flexible feasible aims’ (p.81) of dealing with crises.

Moreover, Collins’ (2004) theory, interaction ritual chain, highlights how social encounters are significant in creating social order, including the importance of emotions in creating a sense of morality (King, 2019). Expanding on this work, King (2019) highlights how shame and embarrassment further explain conformity. Social values are maintained through social interactions and become embedded through everyday rituals. Positive and negative strong feelings maintain social interactions and these everyday rituals, and therefore conformity. Interaction ritual chain theory (King, 2019) is helpful in examining the emotional and moral foundations of conformity towards the government messages of staying at home and protecting the NHS, while such conformity was at times life‐threatening.

This article presents findings from an interview study of people admitted to hospital due to a stroke or heart attack during the COVID‐19 pandemic, drawing on frameworks of candidacy (Liberati et al., 2022) and urgent care work (Turnbull et al., 2019), situating these in a wider socio‐political context through Douglas and Wildavsky’s (1983) perspectives on managing risk and King (2019)’s arguments about the role of social interactions in conformity.

## METHODS

### Recruitment

Twenty‐eight participants were recruited, consisting of 15 participants who had been hospitalised following a heart attack, 11 who had been hospitalised following a stroke, one who had experienced a heart attack and a stroke and one carer of a heart attack survivor. Participants were between 39 and 87 years old, 22 were male and 6 were female. The majority (*n* = 23) self‐identified as White British or White European, three as Asian or mixed‐Asian (*n* = 3), one as Black (West African) (*n* = 1) and one as mixed (White/Black Caribbean) (*n* = 1). In total, 28 interviews were conducted, of which three were paired interviews in which the patient’s spouse participated.

Twenty‐seven participants were identified through cardiac and stroke rehabilitation services in two large acute NHS trusts in the UK Midlands. Participants were eligible if they were admitted to hospital during COVID‐19 lockdown periods between March 2020 and May 2021; were 18 years of age or older; had a confirmed diagnosis of heart attack, stroke or TIA and had been discharged from hospital and completed rehabilitation. Cardiac and stroke rehabilitation teams in both recruitment sites drew random samples of eligible patients from their databases and posted out initial invitation letters, inviting people to contact the research team if they were interested in participating. Table 1 details the recruitment approach and number of recruited participants per site.

**TABLE 1:** Recruitment overview.

Order of dissemination calls	NHS site disseminating recruitment letters	Number of letters and justification	Number of recruited participants
1	Cardiac rehabilitation site 1	200 letters: Original amount intended for all NHS sites and rehabilitation units	*n* = 16 (*n* = 15 heart attack; *n* = 1 heart attack and stroke)
2	Stroke rehabilitation site 1	100 letters: Adjusted down in response to successful recruitment call (1)	*n* = 6
3	Stroke rehabilitation site 2	150 letters: Adjusted up in response to lower response in call (2) to balance sample for stroke and heart attack experience	*n* = 5
4	Cardiac rehabilitation site 2	75 letters: Adjusted down in response to successful recruitment call (1)	None
5	Centre for ethnic health research	Emails and phone calls to seven local cultural organisations and societies, and to local community and faith leaders	*n* = 1
Total			*n* = 28

We selected interviewees from those expressing an interest using a purposive sampling approach to ensure diversity in health condition, age, and gender. We also sought participants from minority ethnic groups through local community groups supported by the Centre for Ethnic Health Research (CEHR). One research participant was successfully recruited via this route.

### Data collection

Given COVID‐19 measures and circumstances, all interviews were conducted remotely, either via telephone or online video call. A topic guide informed by the literature on access to care was developed and used to explore symptom experience and sense‐making, decisions about help‐seeking, routes into hospital and experiences of care (Appendix 1). Written informed consent was obtained in advance from patients and carers. We also included spouses, if requested, at the start or during the interview; spouses were called in to join by the participant when they could not remember important details, or wanted their spouse to recount their experience. In such instances, spouses were informed about the purpose of the interview and verbal consent was recorded. Interviews were conducted by CW (an anthropologist) and JW (an experienced non‐clinical qualitative researcher), and lasted between 30 and 70 min. The interviews were audio‐recorded, transcribed verbatim and anonymised prior to analysis.

### Analysis

Data were analysed using a modified grounded theory approach, in particular, constant comparison (Corbin & Strauss, 2008). Reflective case summaries were written for each interview to help with interpretation and cross‐case comparisons. Members of the research team (CW, CT) repeatedly read initial transcripts, and undertook open coding and note‐taking. Based on this, a preliminary descriptive coding framework was developed and entered into NVivo. Selected transcripts from interviews with stroke and heart attack survivors were coded to further develop and specify the coding framework through team discussion (CW, CT). Once the coding framework was agreed, all interviews were coded applying the final coding framework. We compared across codes to identify and explore themes, and developed descriptive summaries focused on sense‐making of symptoms, candidacy and eligibility, risk and resistance and gender and social networks. We compared across cases to identify similarities and differences in narratives, and iteratively developed a summary table and schematic to reflect common patterns of intersection between experiences of symptoms, judgements of candidacy and the process of navigating emergency care and access to hospital. Contributions to interpretation, and feedback on analysis, were provided by the wider project team (GS, AI, DL, SC, RM).

## FINDINGS

The impact of the pandemic on emergency care help‐seeking varied between individuals, and was highly contingent upon people’s appraisal of their initial symptoms in terms of their meaning, and the level of threat posed. We first outline processes of symptom appraisal and assessment of candidacy, then describe how these intersected with perceptions of messaging and social obligations related to the COVID‐19 pandemic to create forms of moral work. We also discuss the impact of the pandemic on appearance and adjudication by services about eligibility for emergency care.

### Symptom appraisal and sense‐making

The level of perceived threat from symptoms, and participants’ emotional response and perceptions of health risks, shaped subsequent decision‐making about the need for emergency medical attention, and participants’ resultant coping strategies. When participants experienced symptoms that caused them severe pain, or that they recognised as ‘typical’ of stroke or heart attack and led them to experience fear and anxiety, they recognised their legitimate and pressing need for emergency medical attention:

R3 (male, 54, heart attack): The pain started increasing, it was getting really difficult to breathe. My chest was getting tighter and tighter. I started sweating. (…) So, I woke (wife) up and said to her, ‘Look, I’m having serious chest pains and I think I’m having a heart attack. (…) I thought this was, you know, an emergency.

R21 (female, 73, stroke): I thought that I was having a stroke. My mother had had several small strokes, and I just had that feeling that (…) there was something really wrong. (…) I just thought, I need help. Seriously.

In contrast, when individuals experienced their symptoms as worrying but ambiguous, candidacy for emergency care was less obvious. The majority of these individuals appraised their symptoms as presenting something out of the ordinary, but were uncertain about the level of threat posed. Some participants sought advice from family and their social support network in making sense of their symptoms, and establish the legitimacy of seeking help:

R13 (female, 49, heart attack): I felt some tingling sensations in my upper chest area and left arm (…) and a bit of a general tiredness, that I just wanted to sit down. (…) I went downstairs and got dressed as normal and went downstairs and just thought I’d see how I feel and walk around a bit (…). And just, explained the situation to [husband and son] and we both agreed that it wasn’t right and that we should perhaps call someone.

However, a subset of those experiencing ‘non‐typical’ and less severe symptoms made sense of their symptoms in ways that enabled them to discount the threat to health. They coped with uncertainty by attributing symptoms to minor causes, allowing them to avoid anxiety. These coping strategies, had a strongly gendered dimension, being exclusively described by older men. R18 (male, 75, stroke) down‐played his symptoms and attempted to self‐manage, until symptoms escalated to the stage of being severe:

[The beginning of headaches] was the Wednesday. Come the Thursday I felt a little bit worse and a little bit strange. But I thought, you know, take, take some paracetamol it’ll go away. (…) It didn’t go away, no. And then [on Saturday] it hit me, the speech. Which was the first indication, and my facial features shifted to the side. (…) And I’d lost some movement in my right leg and right hand. It was a peculiar feeling, it really was. And I didn’t think for a minute that it was a stroke.

Similarly, R7, male, aged 83 at the incident of a heart attack attributed the pain he experienced to muscle strain. Not recognising the seriousness and downplaying the potential threat, he resorted to previous coping patterns and attempted to self‐manage with additional physical exercise:

I’d never had no problems like this in my life, so I wasn’t sort of expecting it to be a heart attack. (…) When I got one or two of the pains I thought ‐ I’ve always exercised all my life, (…) if I just exercise that area of muscle, it’ll make me feel better. So I did a few press‐ups, one or two other things, which I know now was ridiculous, I shouldn’t have done.

### Moral work and navigating access to services

The impact of the pandemic on help‐seeking was highly variable and contingent on initial sense‐making about symptoms and assessment of candidacy. Despite the unprecedented situation of the COVID‐19 pandemic, people who interpreted their symptoms as presenting an immediate risk to life described seeking help without delay by calling emergency services (or asking or consenting for a partner/relative to do so). For these patients, the risk of mortality was clear and present. An emotional response of fear prevailed in these perceived life‐threatening situations, and candidacy for emergency care was quickly recognised. The majority recalled giving no thought to the risks of COVID‐19, or of conformity to social value expectations, prioritising the risk of mortality over all else. R12 (female, 48, heart attack), fearing she was going to die as the pain level quickly increased from first symptoms to severe pain, called 999:

I’d say about ten minutes later it was just getting worse and worse and worse that I got scared and decided to call an ambulance. (…) I called 999. (…) Because at that point I was scared. I thought that I was going to die. (…) I didn’t even think about COVID at the time to be honest.

R19 (female, 82, stroke), similarly described some concerns about the pandemic, but judged the probable risk to life from her symptoms to outweigh any potential risk from the pandemic:

Well yes, that’s [COVID] at the back of your mind, isn’t it. …The risk of COVID, well it was there, but it wasn’t the pressing need at the time. You balance the risk.

R10 (male, 82, heart attack) described how he and his wife were aware of government messages on how to stay safe from COVID‐19 infection, the pressure on the NHS and the increased risk of contagion in hospitals, but conformity to directives to stay at home was not even considered—once they recognised that his life could be at risk; this acted as a boundary behind which they did not look to question the moral aspects of seeking help. There was no consideration of the moral dimensions of the decision to seek help at a time of extreme demand on the NHS:

R10: I knew about … and we’d been bombarded on the TV by this… by that kind of news but when this happened to me it didn’t even enter my head.

R10’s wife: It didn’t even register.

R10: It didn’t … no, it wasn’t a consideration whatsoever.

As discussed by Douglas and Wildavsky (1983), individuals not only balance risks but also remain flexible to making decisions and taking action contrary to that advocated by their social institutions, by considering immediate risks—in these cases, the immediate risk to life.

However, in cases where candidacy for emergency care was less clear, people’s decisions about whether and how to seek help were complicated by the pandemic and considerations of conformity to social expectations. For the majority of those with ambiguous or mild symptoms, decision‐making about whether and how to seek help involved risk work—balancing perceived threat of symptoms against risk of COVID‐19 if conveyed to hospital—and moral work—assessing the legitimacy, given their particular symptoms, of accessing ambulance and hospital services. Their members of kin acted as a soundboard in sense‐making about symptoms, but also in interpreting the governmental messaging and their eligibility to make use of services: whether they were morally bound to conform to messaging or had a legitimate call on health services. Those who were living with family were more likely to be persuaded to seek medical support. In these cases, reinforcement of their need for help by kin created a moral justification for help‐seeking.

However, these individuals were sensitive to social obligations around conformity to government and media messaging about avoiding over‐burdening the NHS. They had a heightened concern about calling 999, in the context of knowing that the NHS was under pressure due to the pandemic. These morality concerns and fears over non‐conformity (King, 2019) meant that they were under duress to take alternative action. When patients were less clear about the risks posed by their symptoms, they tended to choose lower‐urgency routes for navigating access to care, for example, contacting the 111 service or their general practitioner (GP) rather than calling 999, avoiding the shame of placing additional demand on emergency services contrary to national messaging. This was the case with R13 (female, 49, heart attack), who considered the pandemic both in terms of potential risks to her own health, and in terms of the overwhelming burden it was placing on NHS:

We were quite reluctant to sort of call directly for an ambulance because we were aware that it was just at the point where coronavirus was just kicking off… the feeling was that if you don’t need an ambulance then please don’t call for one. If it was during normal times, I may have called for an ambulance straight away. I think that we just felt (…) reluctant to overwhelm the NHS staff who were already experiencing quite a lot more workload. (…). I was also a bit reluctant to go straight to hospital actually, just with the worry of the virus … being afraid to catch it. (…) I think that was why we thought to phone 111.

Likewise, R25 (male, 70, stroke) reflected on an interplay between desire to conform to government messaging in relation to his own understanding of the seriousness of his condition, moral positioning in terms of avoiding adding to the burden on the health‐care system during the pandemic and heightened risk perception of COVID‐19 during the second wave in winter 2020/2021:

All the adverts on the TV, they say if you think you have a problem and it’s not … it doesn’t warrant a 999 call, dial this number. So that is why I dialled 111. (…) Well it was a second wave of COVID at the time in November 2020, and it was … I’ve had neighbours who have had COVID and one of my close neighbours was seriously [ill] with it (…). As I say, the last thing I wanted to do was to go into hospital where there was much more potential to catch COVID.

R6 (male, 64, heart attack) similarly positioned himself as conforming to government messaging, and making a morally justifiable choice, by choosing a lower‐urgency help‐seeking option—contacting 111 rather than going directly to 999:

Yeah, let 111 decide whether or not I need an ambulance. Because that’s what the television ad(vert) was telling us to do.

The experiences of this group of patients demonstrate how the particular sociocultural and political context of the pandemic shaped interactions, sense‐making and help‐seeking.

As described above, a small group of interviewees initially discounted their symptoms; these participants were the most likely to report delays in seeking help. Their initial symptom appraisal acted as a boundary for making the next decision step: having made sense of their symptoms as not presenting a significant risk to life, they actively avoided help‐seeking. Despite the high‐profile nature of the pandemic, for this group of patients, concerns about conformity to government messaging considerations of moral obligations to avoid burdening the NHS were not features of their decision‐making. Rather it was their interpretation of symptoms as not posing a threat that delayed their help‐seeking.

R18 (male, 75, stroke), who experienced symptoms of a stroke as described above, delayed help‐seeking for four days. His wife noticed the symptoms on the fourth day. He refused to allow his wife to call 999 but was eventually persuaded to call the 111 service himself. As a result of triage, the caller dispatched an ambulance immediately, although when the ambulance arrived, R18 initially resisted conveyance to hospital: ‘*It was only the pressure from my wife that said look*, *you’re gonna have to go*. *(…) I don’t know*, *I don’t like hospitals’*. He argued that the context of the COVID‐19 pandemic was not a factor in his decision‐making about help‐seeking: ‘*I don’t think any of it (COVID) crossed our minds at all’*.

Similarly, R7 (male, 83, heart attack), stoically waited until the following morning to call his GP, notably choosing a low urgency route to seek help. But within minutes of picking up the phone and describing his symptoms, he was told to ‘*just sit tight*, *there’s an ambulance on its way’*. Again, this participant was clear that he was not influenced by COVID‐19 concerns: ‘*I know that there were people not wanting to go to hospital because of the COVID and what was happening*. *But no*, *that never occurred to me’*.

As highlighted in R18’s case above, relatives often had to act on behalf of those who avoided or resisted help‐seeking. For relatives, the moral imperative to ensure the safety of their loved one was more powerful than any moral obligation to society. Relatives had to assess symptom risk themselves, and negotiated with or overruling their loved ones to seek emergency care, such as in the case of R20 (male, 66, stroke):

When my son decided that I was still talking a bit of nonsense, he called an ambulance. I would have stopped him if I could. (…) He came to our house, and I would guess that he called the ambulance, going out to his car and making the call there.

### Appearance and adjudications during the pandemic

When interviewees experienced symptoms that were typical of a stroke or heart attack, or unambiguously life‐threatening, they or their relatives were able to make a clear case for their candidacy when calling for emergency care, and were recognised by services as legitimate candidates for emergency medical attention:

R2 (male, 59, heart attack): ‘I don’t think I was talking to them for more than 30 seconds (…) I literally told them that I started to have chest pains, which got really harder, really more painful and more intense, it spread to my neck, spread to my arms, and then I vomited, and then that’s when she said ‘there’s an ambulance on the way’.

Those who experienced symptoms as ambiguous, and who sought help through lower urgency services, were on the whole subject to rapid assessment and confirmation of their candidacy for immediate emergency medical attention (e.g. in the case of R13 outlined above, ‘*111 did actually call an ambulance’*). But for some participants, the choice of a lower urgency route introduced delays in accessing emergency care, further compounding any delays in deciding to seek help.

Some interviewees also suggested that the switch to telephone appointments by GP practices due to the pandemic had made it more difficult to seek advice and navigate appropriate care when symptoms were ambiguous:

R1 (male, 66, heart attack and stroke): ‘I wish I’d been able to see the doctor rather than talk to him over the phone, because it was a very short conversation. I didn’t feel that I had really put the symptoms over to him and he seemed to say ‘oh well, we can’*t* test you (here) … go to A&E’. Well (I thought) it’s not that bad.’

The heightened salience of COVID‐19, and the prerogative to identify cases of COVID‐19 presenting to urgent care, particularly during the early stages of the pandemic, complicated negotiations about access to help for some participants. R6 (male, 64, heart attack), described how his initial symptoms were incorrectly interpreted by a 111 call‐handler as due to COVID‐19, resulting in a significant delay in getting treatment. This participant described experiencing sudden shortness of breath:

[I] phoned 111 and a doctor phoned me within a couple of minutes. And said ‘ooh, you absolutely must go for a COVID test, if it comes back negative I still want you to isolate because I’m absolutely convinced that you have COVID’.

One day and two negative tests later, R6’s symptoms had worsened. Calling 111 again, and speaking to another call handler, this time an ambulance was sent out. Diagnosed with a heart attack, he underwent surgery putting in stents: ‘*When the surgeon put the camera in my arm and went and had a look*, *he said this damage is two or 3 days old*.’

As such, an additional complicating factor in accessing appropriate emergency care was health professionals’ adjudications in deciding on the type of health‐care provision. When symptoms such as shortness of breath were wrongly attributed to the COVID‐19 virus, delays in accessing appropriate health care created additional harm. Permeability of health care for non‐COVID related illnesses was affected, arguably because of the prevalence of COVID‐19 as a significant threat to the wider public and the social and cultural construction (MacDonald et al., 2016) of COVID‐19 as particularly significant, over other illnesses.

## DISCUSSION

Our findings highlight that the social constructions of COVID‐19 as a threat, and consideration of moral concerns about burdening the health systems in a time of extreme crisis, were interpreted differently by patients depending on their appraisal of their symptoms. These findings reflect existing knowledge about delayed help‐seeking (Jones et al., 2012; Stain et al., 2020), with the added dimension of the context of the pandemic on help‐seeking for serious, non‐COVID‐19 conditions. The pandemic affected those people who experienced symptoms as ambiguous. These individuals either engaged in moral work, balancing desires to conform to national messaging and to avoid overburdening the health system against the uncertain risks posed by their symptoms, and delaying help‐seeking via lower‐urgency services, or avoided and resisted seeking medical assistance until symptoms escalated.

We make important contributions to theorising participants’ illness, navigation and moral work (Turnbull et al., 2019). While the differentiation between severe and mild symptoms in terms of seeking health care advice is highlighted in other studies (Perry et al., 2020; Shokri et al., 2022), our findings highlight symptom appraisal as a key initial stage in decision‐making, that creates path dependency in terms of subsequent decisions about seeking help, and the moral and social dimensions of these. Our findings highlight the arduous challenges connected to moral work. Arguably, government messaging over the risks of COVID‐19 and the advice to ‘Stay Home, Protect the NHS, Save Lives’ (Tan et al., 2021) had implications in delayed help‐seeking for these patients connected to other factors such as fear of contagion and moral obligations towards the NHS, as noted by others such as Nadarajah et al. (2022). Additionally, for these individuals, candidacy, legitimacy and illness identity were compromised with much emphasis being placed on the construction of COVID‐19 as a major threat, and as an unintended consequence, creating ‘collateral damage’ (Pourasghari et al., 2022) and adding to the feelings of uncertainty over sense‐making of symptoms.

Our study considers the importance of socio‐political contexts, and issues of morality and conformity to governmental messaging during the pandemic. While the participants resided in localities which were placed under additional COVID‐19 restrictions, and where fear of contagion and mortality were heightened, political messaging created confusion for those patients whose symptoms were ambiguous, and exposed them to additional risks. Some chose a more ‘flexible’ approach, considering all their options before seeking health‐care support and trying to minimise risks to themselves, kin and communities (Douglas & Wildavsky, 1983); others felt that conforming to the governmental rules overrode their health‐care needs, and were therefore conflicted in what action to take. The latter reluctance is indicative of fears over non‐conformity and not ascribing to common values, as well as the power of governing elite (status group) (King, 2019)—here in the form of governmental messaging—which implied that a common good (the NHS) was threatened by the irresponsible action of individuals. Constructing a narrative to bolster national identity and belonging through repeating a slogan (‘Stay Home, Protect the NHS, Save Lives’), one which invokes emotive reactions towards a national symbol (Ma et al., 2024) and minimises the burden of an already over‐stretched service, may be desirable in times of crisis; however, an all‐encompassing message does not allow for differentiation between vulnerable groups in terms of information on accessing health‐care services. Furthermore, it places considerable strain on patients to act morally and navigate health‐care services, individualising risk (Hanna et al., 2022). Therefore, this study adds to the literature on managing risk during the COVID‐19 pandemic, by highlighting the consequences of governmental messaging which is open to interpretation by vulnerable patients and the complex processes by which decision‐making is reached by taking on ‘urgent care work’ (Turnbull et al., 2019) in times of crisis.

One implication of these findings is that there is a potential value in information that supports sense‐making about ambiguous symptoms—information that goes beyond listing the symptoms of stroke/heart attack and instead describing how people *experience* symptoms of stroke and heart attack. Such an effort was made in February 2022 through the NHS campaign on tackling heart attack myths, encouraging people to seek medical support when they have a feeling that things are ‘really not right’, and are experiencing symptoms that seem unfamiliar (NHS England, 2022). Such an initiative is encouraging in moving away from people listing ‘classic’ symptoms, which are taken as ‘legitimate’ reasons for seeking emergency care.

Our study also highlights the gendered aspects of help‐seeking, with gendered illness identity and related decision‐making influenced by sense‐making about risk and resistance to help‐seeking (Lichtman et al., 2015; Shukla et al., 2022), while kin played a paramount role in taking further action (Hoyer et al., 2020). Our project reinforces evidence on barriers to accessing health care by older men (Mansfield et al., 2008), who were most likely to delay and resist help‐seeking regardless of the context of the pandemic. This adds nuance to the recognition that gender is an important facet of illness identity (Macdonald et al., 2016; Pfeffer, 2004) and suggests that inequalities in accessing emergency care that reflect gender, age and social support may be amplified during times of crisis. This finding invites a different approach to supporting patient groups such as older men, who may be most likely to resist seeking emergency care under any circumstance, encouraging a shift from avoidant to positive or active coping strategies (Leventhal et al., 2016).

The combination of local and national lockdowns, and timing of incidents during the unfolding of the pandemic, played a significant role in decision‐making on seeking emergency care. Lack of salient information on accessing care services for non‐COVID‐19 related illnesses and on procedures in place during hospitalisation, placed some of the participants, and their kin, at significant risk. In times of crises, the complex and at times confusing barriers of urgent health‐care provision (Pope et al., 2019) are further complicated, requiring a re‐framing of expectations of services available and their accessibility, as well as an appraisal of symptoms as permissible to seek such support. Guidance for people about how to navigate the changing landscape of emergency care services during times of crisis would help mitigate delays to urgent help seeking. Such guidance would also minimise navigation and moral work for the patients and their families when interacting with health‐care services.

Our findings have further implications for the conceptualising resilience in health care delivery. Resilience of health‐care systems in the face of shocks, such as the COVID‐19 pandemic, requires actions both to respond to the immediate needs of those affected, and to ensure continued access for all patients to essential services such as emergency and intensive care (Duchek, 2019; Ignatowicz et al., 2023; Kruk et al., 2015; Wears et al., 2015). The adjustments that patients make to their own health care and use of health services in the face of crisis play an important role in resilience (Wiig et al., 2023). The role of the public in scaffolding health system resilience by proactively taking action has been recognised—for example, during the COVID‐19 pandemic, wearing masks to reduce the risk of transmitting infection and avoiding seeking help for minor conditions. O’Hara and colleagues suggest that resilience can be facilitated by providing opportunities for patients to ‘reach in’ to health‐care systems to support the care process, particularly during times of high resource use (O’Hara et al., 2019). Working with patients to develop more nuanced messaging about the moral dimensions of seeking health care, considering immediate risks and longer‐term consequences, may help patients to more safely adjust their approach to help‐seeking. Moreover, our findings suggest that paying attention to how health‐care services ‘reach out’ to vulnerable groups is also crucial for resilience, ensuring those groups seek and access essential services when needed.

Key recommendations from our research study include balancing messaging that emphasises national identity and conformity, with more messaging that is inclusive of vulnerable groups, working with patients to develop more nuanced messaging, providing information about how people *experience* symptoms of serious conditions such as stroke and heart attack to support sense‐making about ambiguous symptoms, providing clear guidance for people about how to navigate the changing landscape of emergency care services during times of crisis and working proactively to reach out to patient groups such as older men to encourage and facilitate help‐seeking.

This study was limited in focus, with participants recruited from two sites in the Midlands area, in England, during the COVID‐19 pandemic. Further research into help‐seeking during other types of crises affecting the accessing of health‐care services, such as health‐care staff strikes, and in other geographical areas could provide further insights. More research into other factors, such as delays in ambulance service reaching patients, or conveyance to hospital, which are beyond the scope of this article, would also reveal further challenges connected to emergency care for those experiencing a stroke or a heart attack.

One of the limitations of this study was the dominance of white British participants over an ethnically diverse population, especially in the wider Midlands area where there is high ethnic diversity. We particularly chose two of the UK’s most ethnically diverse cities and sought targeted recruitment support through the Centre for Ethnic Health Research to pre‐empt such recruitment bias, including disseminating the call for participation in Urdu, Bengali, Gujarati, Hindu and Polish. For Waheed et al. (2015), challenges connected to recruiting ethnically diverse populations relate to a number of factors, such as language barriers, gender, trust, caring responsibilities, employment status, understanding the consent process, lack of culturally competent staff and others. We need to consider the impact of the pandemic on ethnically diverse populations. The pandemic brought much fear and distrust towards the health‐care system, with high numbers of mortality rates for members of ethnically diverse groups recorded from the start of the pandemic (Raleigh, 2022). Nellums et al. (2022) stress the importance of trust building and of using an intersectional approach when engaging with ethnically diverse communities in the UK. Additionally, populations who are vulnerable for reasons of immigration status were also missing from this study. This is an important consideration, as they experience additional barriers in accessing health care (Chase et al., 2017; Koehn, 2009). In trying to capture such experiences, other organisations could be approached, which work directly with refugees and asylum seekers. Further reflection and discussion on how to best support ethnically diverse populations accessing health care, and by extension how to recruit during a health‐care crisis, are much needed. A further limitation was not being able recruit participants with cognitive impairment, speech difficulties and partial memory loss as a result of the incident, whose participation, while posing a challenge to the traditional way of interviewing, especially in the at times necessary remote setting, would have enhanced the sample diversity further.

## CONCLUSION

This study focuses on the experiences of heart attack and stroke survivors in two sites in the Midlands. Analysing their experiences in‐depth has provided important insights into coping strategies and candidacy, illness, moral and navigation work that patients had to undertake during the COVID‐19 pandemic. Our findings describe how sense‐making about symptoms intersects with moral work and conformity to political messages, ultimately reflected in help‐seeking decisions. We add to the literature on risk management and policymaking during crises by advocating for a more nuanced messaging in the context of times of crisis in the NHS, whereby messaging is clear and addresses the needs of diverse urgent care patients and goes beyond the pressures to conform. Although the study was conducted during the pandemic, the findings have wider implications during times of highly publicised strain on the health system, including, for example, winter pressure and staff strikes.

## AUTHOR CONTRIBUTIONS

**Christina Weis**: Investigation; lead, project administration; lead, writing ‐ original draft; lead, **Georgia Spiliopoulos**: Writing ‐ original draft; equal, writing ‐ review & editing; Equal, **Agnieszka Ignatowicz**: Writing ‐ review & editing; equal, **Simon Conroy**: Funding acquisition; equal, writing ‐ review & editing; equal, **Russell Mannion**: Writing ‐ review & editing; equal, **Daniel Lasserson**: Funding acquisition; lead, writing ‐ review & editing; equal, **Carolyn Tarrant**: Conceptualization; lead, funding acquisition; equal, investigation; lead, writing ‐ review & editing; equal.

## CONFLICT OF INTEREST STATEMENT

The authors declare that there is no conflicts of interest.

## ETHICS STATEMENT

Ethical approval was obtained via London Brent Research Ethics Committee, along with HRA approval (20/HRA/5908).

## Supporting information

Supporting Information S1

## Data Availability

The data that support the findings of this study are available on request from the corresponding author [GS]. The data are not publicly available due to their containing information that could compromise the privacy of research participants. The participants of this study did not give written consent for their data to be shared publicly, so due to the sensitive nature of the research supporting data is not available.
