# Supplementary material for: Help‐seeking and access to care for stroke and heart attack during the COVID‐19 pandemic: A qualitative study
Source: Sociol Health Illn. 2024 Sep 20;47(1):e13848. doi: 10.1111/1467-9566.13848 (PMC11684498; doi:10.1111/1467-9566.13848)
Supplement: Supplementary file 1 — Supporting Information S1 [file SHIL-47-0-s001.docx]

Topic Guide

*Introduce self and aim of the study and state approximate length of time interview will take. Seek consent to continue and to audio-record the interview. Let participant know that no personal identifiable data will be recorded and an ID number will be allocated to them. Check throughout that the participant is able to continue, give opportunities for a break if needed.*

**Introductory question**

I believe you recently were/or relative admitted to hospital for a health problem. Probe for what for and when if not already mentioned. How are you feeling now?

- Before you experienced the stroke/heart attack, did you have any underlying conditions (ie chronic illness; other illness...)?
- Had you had any problems accessing healthcare (e.g. seeing your GP or getting routine check-ups) during the pandemic, in the run up to having your stroke/heart attack?

Can you tell me – in your own words – what happened on the day you had the stroke/heart attack?

*Followed by:* Thank you. I will next ask you a few questions which likely will pick up on what you have just told me in more detail.

**Decision-making initial access**

Tell me about the symptoms you/they experienced. What happened when your/their symptoms started and what did you think about the symptoms? Did you think they were serious?

- Where were you? With someone? Alone? What time of the day?

How soon after the symptoms started did you did you seek help/advice? Why? If you delayed, why was this?

Who did you contact about your/your relative’s symptoms? (probe e.g GP, 111, 999 etc.). What happened next?

Thinking about the decision to (call GP/111/999), how did you decide you needed to seek help or advice? Why, what did you consider?

Did you have any concerns about contacting the health service? Why? (Probing: in relation to Covid; if that ever has been the case before)

To what extent were you/your relative worried about having to go to hospital? Why? How did this influence your decisions about what to do next when you had symptoms?

- To what extent were you aware of government messaging or reporting on the TV or in the newspapers about COVID and hospitals? Did you consider this? In what way?
- How did you weigh up the risks to your/your relative’s health? What did you give priority to and why?

What information did you draw on/who did you talk to about whether you/they should contact the health service? Were you aware of any public information about when you should seek help, or about avoiding using healthcare? How did this influence your decision?

How did you make the decision about *which service* to contact? Was it easy to know who to contact? Why?

Overall, how did you feel about contacting the health service for you/your relative’s symptoms during the COVID lockdown?

**Navigating entry**

When you contacted (GP/111/999) how did you explain your/your relative’s symptoms to them? Did you find it relatively straightforward? Why/why not?

- To what extent did you feel that you were taken seriously? What gave you that impression?

How was the decision made about admitting you/your relative to hospital?

Did you/your relative go to the hospital on your/their own? How did you feel about that? Did you have any concern about going there alone/accompanying/them going alone? (Prompts: eligibility, safety, risks, support)

Did you experience any delays between explaining your/your relative’s symptoms and getting to the hospital?

- What do you think contributed to the delays if any?

**Experience in the hospital**

How was the experience of being admitted to hospital?

Did you feel there were any delays in getting the treatment you/your relative needed? At what stage, and why?

Did you worry about any of your individual, dietary, cultural and religious need and whether they would be taken into consideration if you were admitted and if so, did they delay your decision?

Did you feel your individual, dietary, cultural or religious needs were adequately addressed?

Were you/your relative worried about COVID during the hospital admission? What made you feel at risk? Was there anything that made you feel safer?

Can you tell me about your experience of accessing follow-up and rehab clinics

- How have you accessed these (e.g. in person, telephone, online)?
- What has worked well for you in helping you get the right follow up and support?
- Have you had any problems in getting the follow up and support you need? Why?

**Closing**

Thinking about your/your relative’s experience of accessing healthcare for a stroke/heart attack during the pandemic, would you do anything differently? Why/why not?

Do you think the health service could do anything differently, to help people who have a (stroke/heart attack) get treated quickly, even when there are high numbers of people with COVID?

**Demographic questions**

(If not already answered in the interview) Before we end, I would like to ask you a few short (demographic) questions. You can choose not to answer any of these questions, if you prefer.

- When did the incident happen? (Month and year only)

- How old were you at the time of the incident?

What was your living situation at the time of the incident?

- What would you say is your ethnicity?

- What would you say is your gender?

**Thank and close**
